# Reduction of transverse rectal diameter and its effect on bladder dynamics in children with spinal dysraphism

**DOI:** 10.1186/s12894-022-01105-5

**Published:** 2022-09-21

**Authors:** Zoran Radojicic, Sasa Milivojevic, Jelena Milin Lazovic, Ognjen Radojicic, Darko Laketic, Aleksandra Zelenovic, Ivana Dasic, Natasa Milic

**Affiliations:** 1 University Children’s Hospital, Belgrade, Serbia; 2 Faculty of Medicine, Institute for Medical Statistics and Informatics, University of Belgrade, Belgrade, Serbia; 3 Clinic for Gynecology and Obstetrics, Narodni Front, Belgrade, Serbia; 4 Faculty of Medicine, Institute of Anatomy “Niko Miljanic”, Belgrade, Serbia; 5 Department of Internal Medicine, Mayo Clinic, Rochester, USA

**Keywords:** Transverse rectal diameter, Urodynamic findings, Spinal dysraphism

## Abstract

**Introduction:**

To examine the reduction of transverse rectal diameter and its effect on bladder dynamics in children with spinal dysraphism.

**Methods:**

We prospectively evaluated 61 consecutive children with spinal dysraphism, 25 (41%) boys and 36 (59%) girls, aged 4 to 16 years; mean age 9.3 ± 3.8 years, who received bowel management. All children underwent echosonographic measurement of transverse rectal diameter before and after starting bowel management. Also, all the patients had undergone urodynamic studies before and after starting bowel management, with no changes in their urological treatment.

**Results:**

Bowel management caused an decrease in transverse rectal diameter by 56 ± 7.2% (p < 0.001). In addition, a decrease was observed for maximal detrusor pressure by 27.8 ± 7.8% (p < 0.001), leak point pressure by 37.2 ± 4.4% (p < 0.001), and PVR by 36.7 ± 8.0 (p < 0.001). Maximum bladder capacity was significantly increased after bowel management in both non-adjusted (36.4 ± 14.8%; p < 0.001) and adjusted analysis for age (39.4 ± 14.3%, p < 0.001). Detrusor compliance was also increased by 89.2 ± 24.8% (p < 0.001). Female gender and % change of maximal detrusor pressure were significant predictors of transversal rectal diameter change in univariate as well as in multivariate analysis (OR = 10.548, 95% CI 2.309–48.180; p = 0.002 and OR = 1.121, 95% CI 1.009–1.245; p = 0.034).

**Conclusions:**

Decrease in transverse rectal diameter may be useful for bladder function and urodynamic findings in children with spinal dysraphism. Therefore, decrease in transverse rectal diameter should be a supplement to standard urotherapy.

## Introduction

A complicated congenital neuroembryological disorder which has an impact on several organ systems such as bowel function and the bladder is known as Spina bifida. It emerges from an inadequate closure of the neural tube [1].

It is common for children suffering from neurogenic bladder disfunction which has originated due to spina bifida to possess detrusor sphincter dyssynergia (DSD) and detrusor overactivity (DO), as well as an uncontrollable and highly active bladder, incontinence, overactivity in the pelvic floor musculature, and functional bladder outlet obstruction. Correspondingly, the reason for an increased amount of constipation which is often connected with incontinence, is usually the spasm of the pelvic floor and the external anal sphincter [2].

It has been considered common knowledge for a while that constipation can have an impact on lower urinary tract dysfunction in children who do not possess additional urological abnormalities [3, 4].

In contrast, the present available literature emphasizes a scarce amount of papers studying the correlation between bladder and bowel dysfunction in patients with spina bifida, with distinct results being recorded. In order to provide frequent discharge of the colon and faecal continence, alleviate symptoms of constipation, and avoid consistent overdistention of the rectosigmoid, a constant administration of bowel management is crucial [5].

A noninvasive and dependable substitute in evaluating the rectal filling state is the echosonographic measurement of transverse rectal diameter, which could potentially supersede digital rectal examination used during the analysis of children suffering from constipation [5, 6].

This study assess the reduction of transverse rectal diameter and its effect on bladder dynamics in children with spinal dysraphism. The aim of our research is to compare the results of transverse rectal diameter and urodynamic studies in children with spinal dysraphism before and after bowel management.

## Materials and method

A study focused on the observation of children with spinal dysraphism was conducted between 2014 and 2020 which took place at the University Children’s Hospital in Belgrade within the Urology department. During the referred period, 107 children suffering from spinal dysraphism were observed, whereas 79 of them fulfilled the necessary requirements, out of which only 61 agreed to be involved in the investigation. During that time frame, 61 sequential children who had fulfilled the necessary requirements, and agreed to take place in the study with spinal dysraphism were monitored, and were therefore consecutively added in the study. In order to become a part of the study, specific requirements needed to be met by the patients. A period of 65 months, between June 2014 and October 2019 was when the recruitment procedure took place.

Detailed methodology about inclusion and exclusion from the study is published elsewhere [7].

The study encompassed only the spina bifida children in whom constipation was confirmed based on the Roma III criteria and the echosonographic transversal rectal diameter that had to exceed 3 cm [6], while the other patients had to be excluded from the study.

In addition, the study only included patients with the detrusor overactivity (DO) and detrusor sphincter dyssynergia (DSD) which confirmed on the basis of urodynamic testing (cystometry). Finally, a requirement for the inclusion in the study was that the patients had not been on bowel management before, or that they had been in a period of time and quit at least 12 months before the study began.

After the evaluation, all the patients were prescribed bowel management combined with their urological treatment. The observation period was one year.

Detailed methodology about the Bowel management is published elsewhere [7]. In brief, bowel management included diet regimen, retrograde transanal enemas of the order 10–20 ml/kg/day, a maximum of 1 L of physiological solution with an addition of glycerine, and if it was necessary digital rectal stimulation and suppositories (Bisacodyl sup 10 mg). Also, all the patients were prescribed laxatives, those being Polyethylene glycol with additional 0.7 g/kg/day of electrolytes. Two patients had to undergo surgery, so a Malone continent appendicostomy was drawn through the front abdominal wall at the belly button, through which daily antegrade enema. Regular controls for the purpose of evaluating the treatment protocol, i.e. assessing treatment results in all the patients, were carried out every 3 months.

In order achieve the greatest potential conformity with the treatment, a psychologist was present to assist both the children and parents. Following an observation period of one year, the impacts of bowel management with regard to the treatment of constipation were evaluated on the basis of transverse rectal diameter before and after starting bowel management. Every patient went through urodynamic studies prior to, and following the commencement of bowel management, where variations in their urological treatment were not present.

Conditions at the time of the studies did not differ in use of the anticholinergics and the practice of clean intermittent catheterization (CIC), and also no intercurrent operative corrections to bladder or spine had taken place.

Namely, all the patients were prescribed anticholinergic medication therapy (Oxybutynin) 0.2 mg/kg/dose three times daily (adapt according to body weight), and all the patients were also regularly administered CIC every 3 h through a continent vesicostomy, or through a native urethra.

Detailed methodology about the Urodynamic Studies is also published elsewhere [7]. In brief, the urodynamic investigation was conducted according to the International Children’s Continence Society (ICCS) standards [8]. Vesical pressure was recorded with a 6 Fr. double lumen transurethral catheter, abdominal pressures with a 8 Fr. catheter in a small rectal balloon. Activity of the striated pelvic floor muscles was monitored by skin electrodes on the perineum. The bladder was filled with saline warmed to body temperature at a rate of 5–20 ml/min, adjusted to a maximum of 10% of expected bladder capacity for age per minute. Bladder compliance was calculated during filling of the bladder at two thirds of maximum bladder capacity by dividing volume in millilitres and pressure in centimetres water. Maximal cystometric bladder capacity was defined by the maximum volume instilled at 40 cm water pressure, or by the volume instilled before the start of urinary leakage. Detrusor overactivity was diagnosed when repeatedly contractions over 15 cm water pressure are seen. Detrusor leak point pressure (LPP) was considered the least detrusor pressure value in which urine loss occurred in the absence of detrusor contraction or increase in abdominal pressure. The peak detrusor pressure was calculated as the maximal pressure recorded by the cystometry curve during the filling phase in a calm child. Immediately following voiding, post-void residual volume (PVR) was documented right after voiding by catheterization via vesicostomy or transurethrally in patients without vesicostomy with regard to minors who were able to void volitionally. Bladder filling was interrupted when the patient presented with bladder discomfort, strong voiding desire, continuous urinary leak or when the pressure reached 40 cm H_2_O. We also calculated age-adjusted maximal cystometric bladder capacity.

### Transverse rectum ultrasound

During the cystometry via ultrasonography, the measurements and recordings regarding the transversal rectal diameter were obtained. The ultrasound was performed with the patients lying face upwards as explained by Klijn et al. [9] A 7.5 MHz probe was implemented roughly 2 cm above the symphysis, on the abdominal skin. The results were recorded using a reasonably full bladder capacity spanning between 30 and 70% with regard to age, at an angle of approximately 15 degrees below the transverse plane. Two measurements were taken with respect to the diameter of the rectum posterior of the bladder, with an average between the two recordings being determined for each patient. Each patient was questioned in order to determine if they experienced any sort of urge for excretion while the investigation was taking place, and the amount of time in which the patients had defecated before the investigation. If the patient had undergone excretion within the previous two hours, or had experienced an urge to do so throughout the duration of the investigation the procedure would be postponed until the necessary conditions related to intestinal emptying are met.

After one year observation we assessed the effect of bowel management on transverse rectal diameter and urodynamic findings and compared the results before and after bowel management.

Kidney function alongside the upper section of the urinary tract have been monitored through ultrasound prior to, and following bowel therapy. However, a direct evaluation was not present in our study.

#### Side effects

Through the course of each visit, an investigation to determine whether a list of potential side effects of bowel management would occur including; rectal burn, chills, nausea, chemical colitis, flushing, inflammation, dizziness, abdominal or anorectal pain, diarrhea, headaches, sweating, facial flushing, abdominal pain, general discomfort, and bowel perforation with severe and possibly fatal complications. During the visits no side effects were recorded.

#### Statistical analysis

Descriptive statistics were calculated for the baseline demographic and clinical features, as well as treatment outcomes. Continuous variables were presented as means with standard deviations, while categorical variables are presented with numbers and percentages. Differences before and after the treatment were analyzed using Student’s paired T test. Maximal bladder capacity values were adjusted for age according to formula 30 + (30 × age in years) for age up to 12 years, while after 12 age-expected bladder capacity is maintained at 400 ml. Repeated measures ANOVA with gender as covariate were used to assess gender differences in changes of urodynamic parameters. Univariate and multivariate logistic regression was performed to examine predictors of % change in transversal rectal diameter. Mann Whitney test for continuous data as appropriate. Changes in examined variables are presented by box plot graph with dot plot, each presenting individual lmeasurement of urodynamic parameters [10] according to recommendations for data visualization [11]. The level of significance was set at 0.05. Statistical analysis was performed using the IBM SPSS 21 (Chicago, IL, 2012) package.

## Results

An excess of 50% of participants in the study were female, ranging between 4 and 16 years of age. Furthermore, a majority of the patients (over 70%) suffered from aperta spina bifida and an L5 level of lesion, or greater. The most common form of spina bifida was myelomeningocele (over 50%), whereas associated hydrocephalus was presented in beyond half of the cases.

Other relevant characteristics of study population in total and according to gender are presented in Table 1. Indefinite type of spina bifida and high grade VUR were significantly more often observed in girls. Changes of transverse rectal diameter and urodynamic parameters after bowel management are presented in Table 2.

**Table 1:** Baseline demographic and clinical features of study population

Variable	n = 61	Male n = 25	Female n = 36	p
Age, years, mean ± SD	9.3 ± 3.8	8.6 ± 3.6	9.8 ± 3.8	0.241
*Spina bifida, n (%)*				
Aperta	48 (78.7)	17 (68)	31 (86.1)	0.089
Occulta	13 (21.3)	8 (32)	5 (13.9)	
*Level of lesion, n (%)*				
L5 or above	44 (72.1)	18 (72)	26 (72.2)	0.958
S1 or bellow	17 (27.9)	7 (28)	10 (27.8)	
*Type of spina bifida, n (%)*				
Myelomeningocele	35 (57.4)	17 (68)	18 (50)	0.045
Meningocele	6 (9.8)	2 (8)	4 (11.1)	
Lipoma	11 (18)	6 (24)	5 (13.9)	
Indefinite type	9 (14.8)	0 (0)	9 (25)	
Associated hydrocephalus, n (%)	33 (54.1)	16 (64)	17 (47.2)	
VCUG done, n (%)	61 (100)	25 (100)	36 (100)	NA
*VUR confirmed, n (%)*				
Low-grade VUR^*^	3 (4.9)	2 (8)	1 (2.8)	0.05
High-grade VUR^**^	22 (36.1)	5 (20)	17 (47.2)	
Antibiotic prophylaxis^¥^, n (%)	11 (18)	7 (28)	4 (11.1)	0.174
Continent vesicostomy done, n (%)	34 (55.7)			
Uretherovesicostomy^†^	21 (34.4)	5 (20)	16 (44.4)	NA
Appendicovesicostomy	11 (18)	4 (16)	7 (19.4)	
Prepucial vesicostomy	2 (3.3)	2 (8)	0 (0)	
*Treatment administered earlier*				
Oxybutynin + CIC	53 (86.9)	21 (84.0)	32 (88.9)	0.578
Oxybutynin + CIC + bowel management^π^	8 (13.1)	4 (16.0)	4 (11.1)	
Orthopaedic surgical intervention on hip, knee and foot deformities, n (%)	48 (78.7)	7 (28.0)	8 (22.2)	0.606
Bed-ridden patients, n (%)	13 (21.3)	4 (16)	9 (25)	0.399

*VCUG* voiding cystourethrograms;* VUR* vesicoureteral reflux; *NA* not applicable

*I, II and III degree, endoscopically treated with Deflux paste

**IV and V degree, surgically treated with execution of ureteroneocystostomy

^†^From distal end of ureter after executed ureteroneocystostomy

^¥^Administered during the observation period (one-third of therapy dose every evening before bed)

^π^Bowel management quit at least 12 months before the study began

**Table 2:** Changes of transverse rectal diameter and urodynamic parameters after bowel management

	Before	After	p
Transverse rectal diameter	49.4 ± 8.4	21.6 ± 4.6	< 0.001
Maximum bladder capacity	178.8 ± 93.2	231.5 ± 98.6	< 0.001
Decrease in maximal detrusor pressure	61.6 ± 14.4	44.8 ± 12.7	< 0.001
Detrusor compliance	3.1 ± 2	5.7 ± 3.3	< 0.001
Leak point pressure	58.3 ± 11.6	36.5 ± 7.6	< 0.001
Post-void residual volume	164.9 ± 58.9	106.7 ± 47	< 0.001

**Fig. 1:**
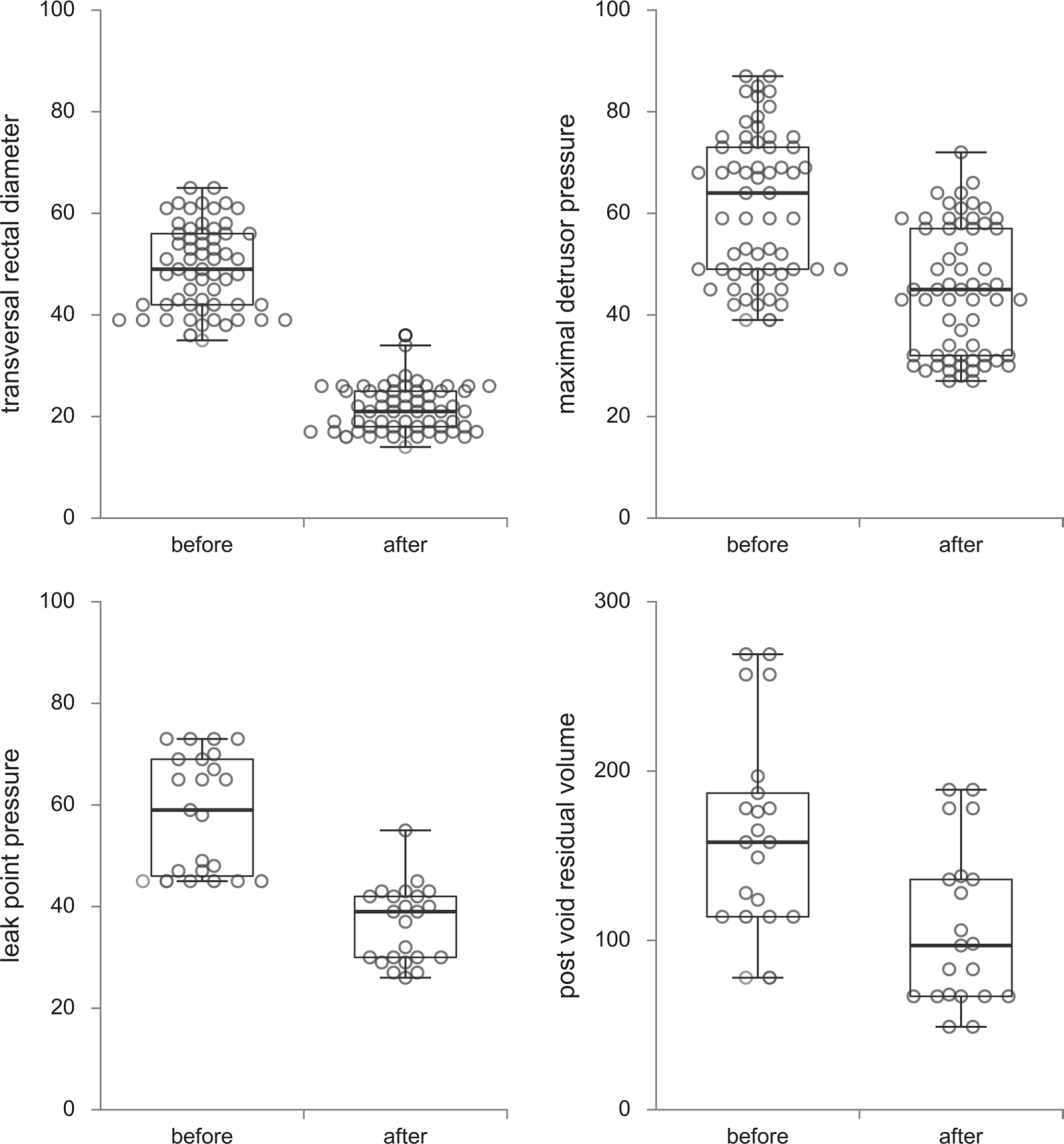
Changes of transverse rectal diameter and urodynamic parameters after bowel management

Bowel management caused an decrease in transverse rectal diameter by 56 ± 7.2% (p < 0.001) (Fig. 1). We have examined transversal rectal diameter according to gender. There was no significant difference in transversal rectal diameter before bowel management. In contrast, after bowel management there was a significant difference in transversal diameter, diameter was lower in girls and percent change of diameter was higher in girls are presented in Table 3. In addition, we have examined transversal rectal diameter according to lesion level. There was a significant difference in transversal diameter before bowel management, diameter was lower in S1 and bellow lesion, while there was no difference between transversal diameter after as well as % change of transversal diameter according to lesion level are presented in Table 4.

**Table 3:** Changes of transverse rectal diameter according to gender

	Male	Female	p
Transversal diameter before	50 ± 6.9	49.1 ± 9.3	0.692
Transversal diameter after	23.2 ± 3.2	20.5 ± 5.1	0.020
% change of transversal diameter	− 52.7 ± 8.1	− 58.2 ± 5.6	0.003

**Table 4:** Changes of transverse rectal diameter according to lesion level

	L5 or above	S1 or bellow	p
Transversal diameter before	51.1 ± 8.2	45.1 ± 7.3	0.011
Transversal diameter after	22.2 ± 4.7	20.2 ± 4	0.141
% change of transversal diameter	− 56.4 ± 7.6	− 55 ± 6.1	0.527

**Fig. 2:**
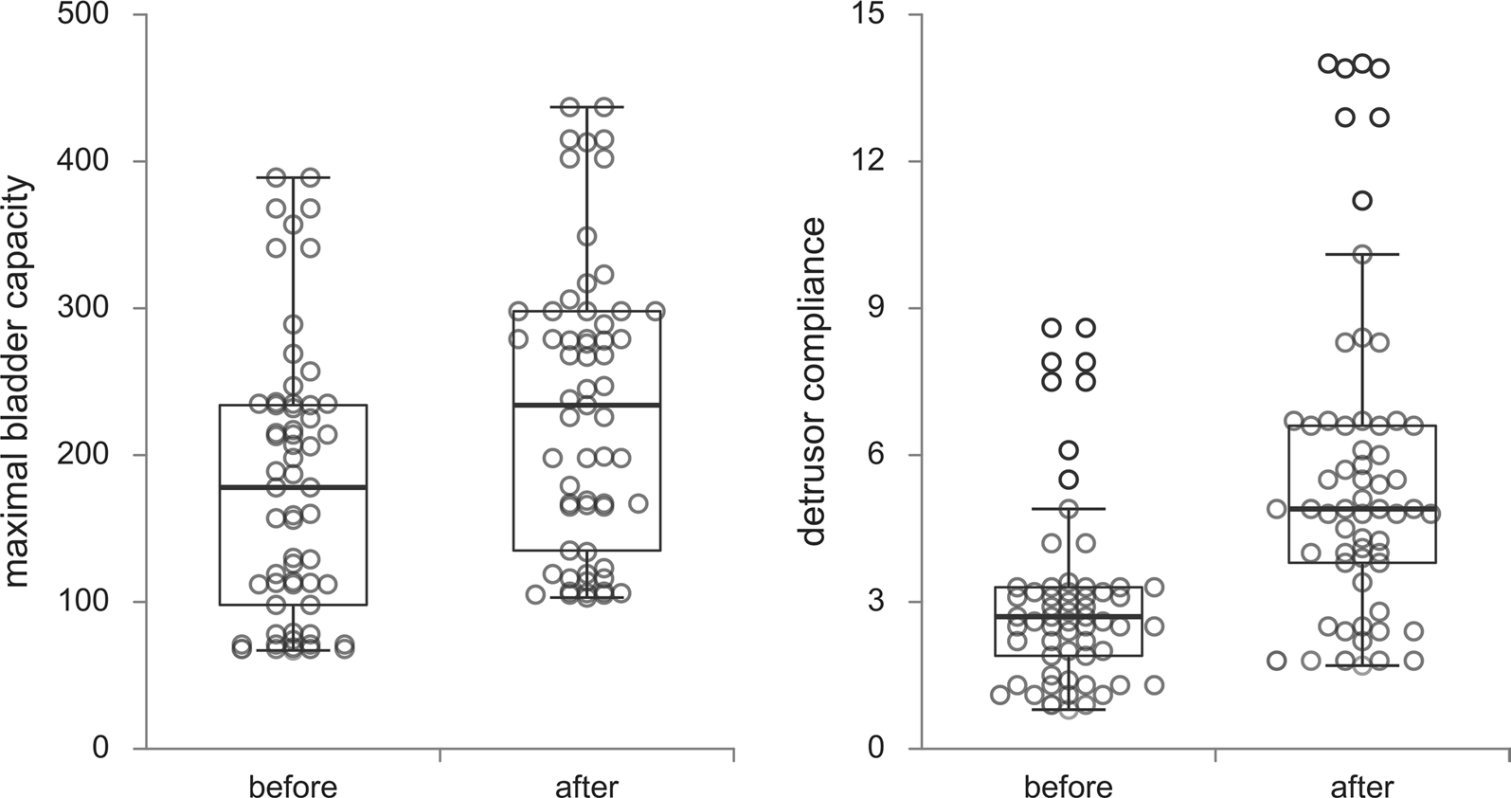
Changes of urodynamic parameters after bowel management

In addition, a decrease was observed for maximal detrusor pressure by 27.8 ± 7.8% (p < 0.001), leak point pressure by 37.2 ± 4.4% (p < 0.001), and PVR by 36.7 ± 8.0 (p < 0.001) (Fig. 1). Maximum bladder capacity was significantly increased after bowel management in both non-adjusted (36.4 ± 14.8%; p < 0.001) and adjusted analysis for age (39.4 ± 14.3%, p < 0.001). A significant interaction was observed for gender and maximal bladder capacity (p = 0.023), as girls had significantly higher maximal bladder capacity in the beginning of treatment, compared with male. Detrusor compliance was also increased by 89.2 ± 24.8% (p < 0.001) (Fig. 2). Results of univariate logistic regression with transversal rectal diameter as depended variable are presented in Table 5. Female gender and % change of maximal detrusor pressure were significant predictors of transversal rectal diameter change in univariate as well as in multivariate analysis (OR = 10.548, 95% CI 2.309–48.180; p = 0.002 and OR = 1.121, 95% CI 1.009–1.245; p = 0.034).

**Table 5:** Univariate logistic regression for percent change of transversal rectal diameter

	B	S.E.	Sig.	OR	95% CI for OR	
					Lower	Upper
Gender	2.157	0.725	0.003	8.643	2.086	35.808
Age	− 0.056	0.082	0.497	0.946	0.805	1.111
% max bladder capacity	0.022	0.022	0.314	1.022	0.980	1.066
% max detrusor pressure	0.097	0.047	0.040	1.102	1.004	1.209
% compliance	− 0.019	0.012	0.133	0.981	0.958	1.006
%leak point pressure	− 0.285	0.153	0.063	0.752	0.557	1.015
% PVR	0.120	0.107	0.262	1.127	0.914	1.389

Also, we have examined changes of urodynamic parameters and transversal diameter with lesion level interaction. We used repeated measures ANOVA with factor interaction. Significant interaction was found in changes of transversal rectal diameter and lesion level (p = 0.029). We have found no significant difference in changes of urodynamic parameters with lesion level interactions (maximum bladder capacity * lesion level p = 0.402; maximal detrusor pressure*lesion level p = 0.281; compliance * lesion level p = 0.245; leak point pressure * lesion level p = 0.533, PVR* lesion level p = 0.117).

We have found no significant difference between percent changes of transversal diameter and percent changes of urodynamic findings.

A great degree of compliance was evident by the patients throughout the observation period with regard to the treatment protocol with the exception of a total of three patients with follow ups less than a year as a result of the prior therapy discontinuation.

## Discussion

It has been known for a long time now how constipation can impact urinary tract infections (UTIs), lower urinary tract function, and urinary incontinence in children who do not possess other urological deformities [3, 4].

Many theories exist clarifying the correspondence between lower urinary tract dysfunction and constipation, such as the anatomical proximity of the bladder and urethra to the rectum and their typical embryological origin and comparable innervation [12].

A potential alternate explanation might be that when a significant amount of fecal matter is retained in the rectum and sigmoid colon, compression of the bladder is caused inclusive of the neck, therefore substantially impacting bladder capacity and contractility (via decreasing functional capacity through stimulation of a rise in bladder contractility) [13], or absolutely block bladder activity [14].

In addition, one other way in which this may be explained is through the robust link between the bladder and bowel in the central nervous system [15].

Regardless of the various disputable arguments surrounding the predictive value of transverse rectal diameter in the analysis of functional constipation, this evaluation is being portrayed as an extremely important tool with respect to diagnosing functional constipation in children [16, 17].

It has been expressed by a number of authors that digital rectal examination could potentially be substituted by identifying transverse rectal diameter as an accurate parameter, and at the forefront of investigating functional constipation [9, 18, 19].

Moreover, a significant deviation has been observed when analyzing the cut-off point utilized in establishing a connection between functional constipation and increased rectal diameter, with the proposed cut-off points spanning from 2.4 to 4 cm [6, 9, 20, 21].

Nevertheless, the available literature at present shows that there are a limited number of papers studying the relationship between bladder dysfunction and bowel dysfunction in spina bifida patients, with conflicting results.

Among the very few studies available on the subject, there is one paper by Kort et al. which resulted in no statistically pertinent alterations being determined in urodynamic parameters once bowel management in patients with spina bifida was administered. With reference to bladder compliance, bladder capacity, bladder instability, and leak-point pressure, no major changes were recorded [2].

However, a study by Cameron et al. which investigated the intensity of bowel dysfunction in patients with neurogenic bladder comprised of completely contradictory findings. The direct correspondence between bowel symptom scores with lower urinary tract symptoms and bladder incontinence manifestations, outlines the importance for urologists to acknowledge urinary issues in conjunction with bowel dysfunction [22].

A recent study of ours precisely demonstrated how the administration of bowel management could be helpful with regard to urodynamic discoveries and bladder function in children suffering from spinal dysraphism who have detrusor sphincter dyssynergia and overactivity [7].

The prevalence of neurogenic bowel complications with reference to the subjects participating in our investigation inclusive of fecal incontinence, abdominal pain, constipation, and soiling were 89% and does not differ significantly from the current literature.

But so far we did not examined reduction of transverse rectal diameter and its effect on bladder dynamics in children with neurogenic bowel and bladder dysfunction.

For that very reason, the aim of the present study was to test the reduction of transverse rectal diameter and its effect on bladder dynamics in children with spinal dysraphism with DO and DSD.

Owing to the administered bowel management in our present study, we caused an decrease in transverse rectal diameter by 56 ± 7.2%.

Bowel management was equally effective in our patients regardless of the type of lesion, although the transversal diameter was lower in S1 and bellow lesion before the implementation of bowel management. Also, after bowel management transversal diameter was lower in girls and percent change of diameter was higher in girls, and as a possible reason for that we emphasize better compliance with therapy in girls, that we will consider in future studies.

Consequently, we were able to demonstrate how administering bowel management attained significantly more control over constipation which was indicated in echosonographic transversal rectal diameter < 3 cm, in 59 (96.7%) of the patients with spina bifida.

In addition, the results of the present study clearly indicate that decrease in transverse rectal diameter may noticeably changes the urodynamic variables of neurogenic bladder dysfunction in children with spinal dysraphism with DO and DSD.

The effect was confirmed by an increase in maximum bladder capacity, a decrease in maximal detrusor pressure and an increase in bladder compliance. There was also significant reductions in leak point pressure and significant reductions in PVR in our patients who could achieve spontaneous voiding. A significant interaction was observed for gender and maximal bladder capacity (p = 0.023), as girls had significantly higher maximal bladder capacity in the beginning of treatment, compared with male. Also, female gender and % change of maximal detrusor pressure were significant predictors of transversal rectal diameter change. Therefore, we conclude that in children with spinal dysraphism with neurogenic bowel and bladder dysfunction, decrease in transverse rectal diameter may be useful for bladder function and urodynamic findings. It is difficult for us to say to what extent and through which exact mechanism decrease in transverse rectal diameter may be useful for bladder function in our patients, but we will seek answers to these questions in future urodynamic testing.

However, bowel management is a big challenge. Its beneficial effects last only while the therapy is carried out, which can be seen in the example of our 12 patients in whom bowel management was previously implemented, then discontinued on their own initiative, and in whom there was a complete return of constipation due to discontinuation of therapy.

Bowel management does not exclude standard urotherapy, but is a supplement to standard urotherapy, which may be useful for bladder function and urodynamic findings.

The limitations of the present study include the small number of studied patients, and the fact that the study was not randomized. Due to the specific way in which our study is constructed, it is impossible to establish whether or not the effect discovered (urodynamic change) is the outcome solely from the resolution of transverse rectal diameter or whether with the passage of time or through the use of other potential treatments like anticholinergics, and/or others similar, have led to the improvement of the participants. Moreover, this is a drawback that must be taken into consideration for future investigations. It is also a disadvantage that we will consider in future studies. The additional limitations of the study are that we did not investigate the kidney function and morphology before and after bowel management. These limitations should be taken into consideration before further research is conducted.

## Conclusion

Decrease in transverse rectal diameter may be useful for bladder function and urodynamic findings in children with spinal dysraphism. Therefore, decrease in transverse rectal diameter should be a supplement to standard urotherapy.

## Data Availability

The datasets used and/or analyzed during the current study are available from the corresponding author on reasonable request.

## References

[1] Johnston AW, Wiener JS, Purves JT (2020). Pediatric neurogenic bladder and bowel dysfunction: will my child ever be out of diapers?. Eur Urol Focus.

[2] De Kort LM, Nesselaar CH, van Gool JD (1997). The influence of colonic enema irrigation on urodynamic findings in patients with neurogenic bladder dysfunction. Br J Urol.

[3] Dohil R, Roberts E, Verrier Jones K (1994). Constipation and reversible urinary tract abnormalities. Arch Dis Child.

[4] Loening-Baucke V (1997). Urinary incontinence and urinary tract infection and their resolution with treatment of chronic constipation of childhood. Pediatrics.

[5] Velde SV, Biervliet SV, Bruyne RD (2013). A systematic review on bowel management and the success rate of the various treatment modalities in spina bifida patients. Spinal Cord.

[6] Burgers R, de Jong TP, Benninga MA (2013). Rectal examination in children: digital versus transabdominal ultrasound. J Urol.

[7] Milivojevic S, Milic N, MilinLazovic J (2020). The influence of bowel management on urodynamic findings in spina bifida children with detrusor overactivity and detrusor sphincter dyssynergia. J Pediatr Urol.

[8] Austin PF, Bauer SB, Bower W (2016). The standardization of terminology of lower urinary tract function in children and adolescents: update report from the standardization committee of the International Children’s Continence Society. Neurourol Urodyn.

[9] Klijn AJ, Asselman M, Vijverberg MAW (2004). The diameter of the rectum on ultrasonography as a diagnostic tool for constipation in children with dysfunctional voiding. J Urol.

[10] Weissgerber TL, Savic M, Winham SJ (2017). Data visualization, bar naked: a free tool for creating interactive graphics. J Biol Chem.

[11] Weissgerber TL, Winham SJ, Heinzen EP (2019). Reveal, don’t conceal: transforming data visualization to improve transparency. Circulation.

[12] Bower WF, Yip SK, Yeung CK (2005). Dysfunctional elimination symptoms in childhood and adulthood. J Urol.

[13] Burgers R, Liem O, Canon S (2010). Effect of rectal distention on lower urinary tract function in children. J Urol.

[14] Miyazato M, Sugaya K, Nishijima S (2004). Rectal distention inhibits bladder activity via glycinergic and GABAergic mechanisms in rats. J Urol.

[15] Franco I (2011). The central nervous system and its role in bowel and bladder control. CurrUrol Rep.

[16] Singh SJ, Gibbons NJ, Vincent MV (2005). Use of pelvic ultrasound in the diagnosis of megarectum in children with constipation. J Pediatr Surg.

[17] Berger MY, Tabbers MM, Kurver MJ (2012). Value of abdominal radiography, colonic transit time, and rectal ultrasound scanning in the diagnosis of idiopathic constipation in children: a systematic review. J Pediatr.

[18] Drossman DA, Hasler WL (2016). Rome IV–functional GI disorders: disorders of gut-brain interaction. Gastroenterology.

[19] Joensson IM, Siggaard C, Rittig S (2008). Transabdominal ultrasound of RM as a diagnostic tool in childhood constipation. J Urol.

[20] Karaman A, Ramadan SU, Karaman I (2010). Diagnosis and follow-up in constipated children: should we use ultrasound?. J Pediatr Surg.

[21] Hatori R, Tomomasa T, Ishige T, Tatsuki M, Arakawa H (2017). Fecal retention in childhood: evaluation on ultrasonography. Pediatr Int.

[22] Cameron AP, Rodriguez GM, Gursky A (2015). The severity of bowel dysfunction in patients with neurogenic bladder. J Urol.

